# Remediation of Cadmium-Contaminated Soil Using Sodium Alginate-Embedded Rectorite and Organoamine-Modified Rectorite Microspheres

**DOI:** 10.3390/molecules31111851

**Published:** 2026-05-28

**Authors:** Xuan Xia, Qinhan Ye, Yang Xiao, Hanjun Wu, Xinhong Qiu

**Affiliations:** School of Chemistry and Environmental Engineering, Wuhan Institute of Technology, Wuhan 430079, China

**Keywords:** rectorite, alginate microspheres, amine functionalization, Cd(II) immobilization, soil remediation

## Abstract

Cadmium (Cd) contamination in agricultural soils poses serious risks to ecosystem health and food safety, highlighting the urgent need for efficient and environmentally stable immobilization materials. In this study, sodium alginate-based rectorite microspheres (REC beads) and triethylenetetramine-modified rectorite microspheres (TETA-REC beads) were fabricated and applied for the immobilization of Cd(II) in contaminated soils. Structural characterization confirmed that the ionic cross-linking encapsulation process preserved the layered structure of rectorite, while TETA modification introduced abundant amino functional groups that enhanced the interaction between the material and Cd(II). Immobilization experiments demonstrated that both microspheres exhibited rapid and stable Cd(II) passivation performance, with TETA-REC beads showing significantly higher efficiency. The maximum removal efficiencies of water-soluble and available Cd(II) reached 83.87% and 93.33%, respectively. Sequential extraction analysis revealed that the microspheres effectively transformed labile Cd fractions, including exchangeable and water-soluble forms, into more stable species. Mechanistic investigations indicated that Cd immobilization was governed by a synergistic combination of interlayer ion exchange, physical adsorption within the porous alginate structure, and coordination complexation between Cd(II) and amino groups introduced by TETA. Furthermore, microbial community analysis showed that the remediation process promoted the recovery of soil microbial diversity and metabolic functions. The developed TETA-REC microspheres provide a promising strategy for the efficient immobilization of Cd in contaminated soils and offer potential for the sustainable remediation of heavy metal-polluted farmland.

## 1. Introduction

Cadmium (Cd) is a widely used heavy metal in industrial activities such as the production of specialty steels, alloys, electroplating materials, and textiles [1]. However, intensive industrialization has resulted in the continuous release of cadmium into the environment, where it commonly exists in soluble inorganic forms and can readily accumulate in water and soil systems [2]. Among its various chemical species, Cd(II) is the most prevalent and toxic form [3]. It has been classified as a human carcinogen by the International Agency for Research on Cancer (IARC) and the United States Environmental Protection Agency (US EPA) [4]. Long-term exposure to cadmium may cause severe health problems [5,6], including kidney dysfunction, bone demineralization, and osteoporosis. Consequently, cadmium contamination in soils has become a global environmental concern [7,8]. The accumulation of Cd in agricultural soils not only deteriorates soil quality but also leads to elevated Cd concentrations in crops, which can subsequently enter the human body through the food chain [9]. Therefore, developing efficient and environmentally sustainable strategies to reduce the ecological risk and bioavailability of Cd(II) in soils is of great importance for environmental protection and food safety [10].

Among the available remediation technologies, chemical passivation has attracted considerable attention due to its operational simplicity, relatively low cost, and rapid effectiveness. In this approach, immobilizing agents are introduced into contaminated soils to transform bioavailable heavy metals into more stable and less mobile forms. Natural clay minerals are particularly promising candidates for such applications because of their abundance, low cost, and environmental compatibility [11,12]. These minerals possess high surface areas and abundant active sites [13,14], enabling the immobilization of heavy metal ions through mechanisms such as ion exchange, surface adsorption, and complexation [15].

Rectorite (REC), a characteristic clay mineral widely distributed in China, particularly in Hubei Province [16], has attracted increasing attention in the field of environmental remediation. Its unique layered structure consists of alternating illite and montmorillonite layers arranged in a regular 1:1 ratio [17,18]. The presence of montmorillonite layers endows rectorite with relatively high interlayer cation exchange capacity and swelling properties [19], which facilitate interactions with metal ions [20]. In addition, the surface of rectorite contains abundant silanol groups (Si–OH) and Brønsted–Lewis acid sites that can interact with functional molecules or polymer chains, thereby enhancing the stability of composite materials. Owing to these structural characteristics, rectorite has been widely investigated as an adsorbent or composite component for the removal of heavy metals. For example, Wang et al. [21] prepared a TiO_2_-REC composite material for the removal of heavy metals from acidic wastewater. Gao et al. [22] further developed magnetic rectorite for the efficient adsorption of Pb(II) from wastewater, enabling an integrated process of adsorption, separation, and reuse. However, natural rectorite primarily immobilizes heavy metals through cation exchange and physical adsorption, and its chemical complexation ability toward certain metal ions remains limited.

To overcome this limitation, organic functionalization has been widely employed to enhance the affinity of clay minerals toward heavy metals [23]. In particular, the introduction of organic amine molecules containing nitrogen-based coordination groups (e.g., –NH_2_) can significantly improve the chemical binding capacity of clay materials. The intercalation of organic molecules can regulate the interlayer spacing and surface chemical properties of clay minerals, thereby improving their dispersion and surface reactivity. Moreover, amino groups are able to form stable coordination bonds with heavy metal ions, which greatly enhances immobilization selectivity and stability. Previous studies have demonstrated the effectiveness of such modification strategies. For example, Xu et al. [24] further modified rectorite using photoactive covalent organic framework (COF) polymers for the treatment of Cr(T) in solution, successfully achieving both Cr(VI) reduction and chromium removal. Nevertheless, the practical application of powdered clay minerals in soil remediation still faces several challenges. Fine particles are easily dispersed within soil matrices, making their recovery and reuse difficult and thereby limiting their engineering applicability [25]. To address this limitation, encapsulating functional clay materials within polymer matrices to form granular composites has emerged as a promising strategy.

Sodium alginate (SA), a natural polysaccharide derived from brown algae [26], has been widely used in environmental and biomedical applications due to its excellent biocompatibility, gel-forming ability, and facile crosslinking characteristics [27,28]. In the presence of Ca^2+^ ions, SA can form a typical “egg-box” structure through ionic crosslinking [29,30], resulting in a stable three-dimensional hydrogel network with good mechanical strength and shaping ability. Recently, SA-based hydrogel microspheres have shown great potential in environmental remediation. For example, Liu et al. [31] developed peptide-coated SA hydrogel microspheres for selective Ga adsorption, while Chen et al. [32] constructed magnetic lanthanum-doped clay/SA composite hydrogel beads for efficient phosphate removal from wastewater. These studies demonstrate that SA can significantly enhance the structural stability, operability, and reusability of composite materials in complex environmental systems.

Based on this concept, the present study aims to fabricate novel microspherical passivation materials by encapsulating rectorite (REC) and triethylenetetramine-modified rectorite (TETA-REC) within a sodium alginate matrix. The transformation of powdered REC into large-particle microspheres is expected to improve material handling, separation efficiency, and reusability during soil remediation processes. The prepared REC microspheres were applied to Cd(II)-contaminated soils, and systematic passivation experiments were conducted under different treatment times. The concentrations of water-soluble and available Cd(II) in soil were used as key indicators to evaluate the immobilization performance and remediation efficiency of the developed microsphere materials.

## 2. Results and Discussion

### 2.1. Characterization of REC Microspheres

**Figure 1:**
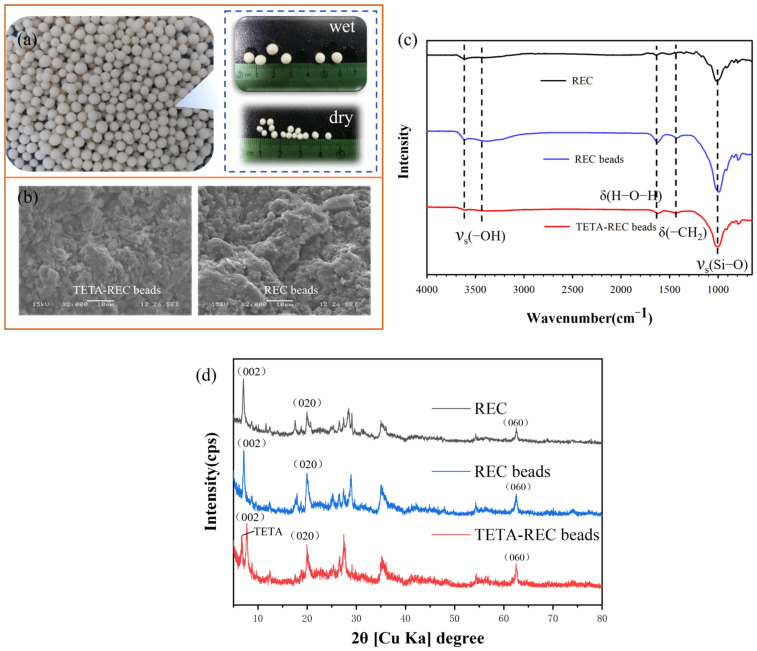
(**a**) The appearance of wet and dry REC beads. (**b**) The SEM of dry REC beads. (**c**) The FTIR spectrum of REC beads and TETA-REC beads. (**d**) The XRD results of REC beads and TETA-REC beads.

Figure 1 presents the XRD patterns, FTIR spectra, photographs before and after drying, and SEM images of the REC microspheres. Figure 1a,b show the physical appearance of REC microspheres before drying and their microscopic morphology after drying, respectively. The wet REC microspheres exhibited relatively regular spherical shapes with soft texture, low elasticity, and diameters generally ranging from 5–6 mm. After drying, the REC microspheres became irregular in shape with obvious shrinkage and surface depressions. Meanwhile, their hardness increased while elasticity remained low, and their diameters decreased to approximately 2–3 mm. The reduction in microsphere volume was mainly attributed to the evaporation of internal moisture during the drying process, which caused the originally swollen REC structure to shrink due to water loss. Scanning electron microscopy revealed that the surface of the dried REC microspheres contained numerous pores and gaps. Compared with unmodified REC beads, the surface of TETA-REC beads appeared relatively smoother and more compact, suggesting improved dispersion and more homogeneous distribution of the modified REC within the alginate matrix. This compact surface morphology did not necessarily indicate blockage of internal diffusion pathways, as the microspheres still retained internal pores and channels generated during gelation and drying processes. Moreover, the enhanced Cd(II) immobilization performance of TETA-REC beads was mainly attributed to the introduction of amino functional groups, which provided additional coordination sites for Cd(II).

Figure 1c shows the Fourier transform infrared (FTIR) spectra of REC beads and TETA-REC beads. In the spectra, the region at 3300–3700 cm^−1^ corresponds to the stretching vibration of hydroxyl (–OH) groups. The characteristic peak at 3614 cm^−1^ is attributed to the stretching vibrations of Si–OH and Al–OH groups, while the broad absorption band around 3400 cm^−1^ corresponds to the stretching vibration of hydrogen-bonded –OH groups. In addition, the peak near 1635 cm^−1^ represents the bending vibration of H–O–H, indicating the presence of adsorbed water in the REC microspheres. Compared with the original REC sample, the intensities of both the stretching and bending vibration peaks of water were enhanced in REC beads and TETA-REC beads, suggesting that part of the water was retained within the microspheres during the bead-forming process. The strong absorption band at 980–990 cm^−1^ is assigned to the stretching vibration of Si–O bonds. Furthermore, both types of microspheres exhibited a characteristic peak at approximately 1456 cm^−1^ corresponding to the –CH_2_ bending vibration, which was absent from the original REC sample. This peak is likely derived from the –CH_2_ groups present in sodium alginate molecules.

The two types of microspheres were ground into powder prior to XRD characteri-zation, and the results are shown in Figure 1d. As shown in the XRD pattern, the REC microspheres exhibited characteristic reflections at 2θ values of 7.09°, 19.98°, and 62.59°, corresponding to the (002), (020), and (060) crystal planes of rectorite, respectively. It should be noted that the diffraction peak near 7° corresponds to the (002) basal reflection of rectorite rather than the primary (001) reflection. Rectorite is a regularly interstratified clay mineral characterized by a periodic superlattice structure, and its fundamental (001) reflection generally appears in the low-angle region at approximately 3.5–4.0° (d_001_ ≈ 2.2–2.5 nm). The peak observed at 7.09° therefore represents the higher-order (002) reflection corresponding to approximately half of the superlattice periodic spacing. According to Bragg’s equation (2dsinθ = nλ), the spacing calculated from the diffraction peak at 2θ = 7.09° was approximately 1.24 nm, corresponding to the d_002_ value of rectorite. Since rectorite is a regularly interstratified layered clay mineral, the actual basal spacing can be obtained from d_001_ = 2d_002_, yielding a d_001_ value of approximately 2.48 nm, which is consistent with the characteristic interlayer spacing reported for rectorite. After alginate encapsulation, d_002_ increased to 1.26 nm, which may be attributed to interactions between sodium alginate functional groups and exchangeable interlayer cations during Ca^2+^-induced cross-linking, resulting in slight expansion of the layered structure. After TETA modification, the d_002_ spacing increased to 1.32 nm, corresponding to a d_001_ value of approximately 2.64 nm. The increase in basal spacing suggests that TETA molecules were successfully intercalated into the interlayer region of rectorite, resulting in expansion of the layered structure. This phenomenon is consistent with the typical intercalation behavior of polyamine molecules in layered clay minerals and indicates strong interactions between TETA molecules and the clay interlayers.

### 2.2. Effect of Time on Cd(II) Passivation

**Figure 2:**
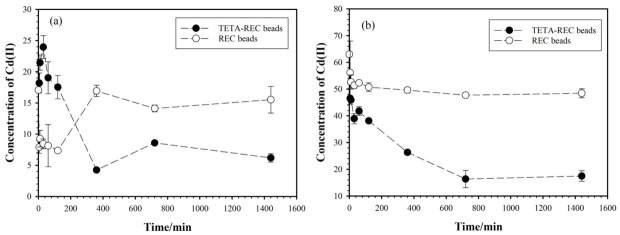
The changes in (**a**) water-soluble state and (**b**) available state with the increase in immobilization time (concentration of Cd(II)-contaminated soil: 1000 mg/kg, dosage of beads: 0.05 g).

Figure 2 illustrates the variation in water-soluble and available Cd(II) concentrations in contaminated soil with passivation time. In soil with an initial Cd(II) concentration of 1000 mg/kg, the water-soluble Cd(II) concentration in the control group was 17.07 mg/L, while the available Cd(II) concentration extracted using 0.01 mol/L CaCl_2_ was 63.06 mg/L. The passivation process tended to stabilize after 24 h. As shown in the figure, after the addition of REC beads, the water-soluble Cd(II) concentration rapidly decreased to 7.91 mg/L within 5 min and reached a relatively stable level within 2 h. However, the concentration increased again after 6 h, indicating partial re-release of Cd(II), and finally reached 15.52 mg/L at 24 h. This phenomenon may be related to the gradual release of Ca^2+^ from the interlayer of attapulgite in the microspheres, which could promote ion-exchange reactions and lead to the release of Cd(II) under continuous mixing conditions. In contrast, TETA-REC beads exhibited significantly better passivation performance. During the initial 30 min of passivation, the water-soluble Cd(II) concentration briefly increased to 23.96 mg/L, likely due to the rapid release of Ca^2+^ from the microspheres. However, the strong complexation between TETA molecules and Cd(II) rapidly immobilized Cd(II), ultimately reducing its concentration to only 4.24 mg/L after 24 h. This result indicates that TETA possesses strong coordination affinity and complexation stability with Cd(II). For the passivation of available Cd(II), both REC beads and TETA-REC beads showed certain effectiveness. Within 24 h, REC beads reduced the available Cd(II) concentration from 63.06 mg/L to 48.45 mg/L, whereas TETA-REC beads further decreased it to 17.46 mg/L. This substantial difference reflects the stronger stabilization capacity of TETA-REC beads toward Cd(II). The enhanced performance may be attributed to the introduction of additional complexation sites by TETA molecules, which significantly improves their chelation and stabilization ability toward Cd(II). In contrast, the immobilization of Cd(II) by unmodified REC mainly relies on physical adsorption and ion-exchange processes, resulting in relatively limited long-term stabilization capacity.

### 2.3. Effect of Different Initial Concentrations of Cd(II) on the Passivation of REC Microspheres

**Figure 3:**
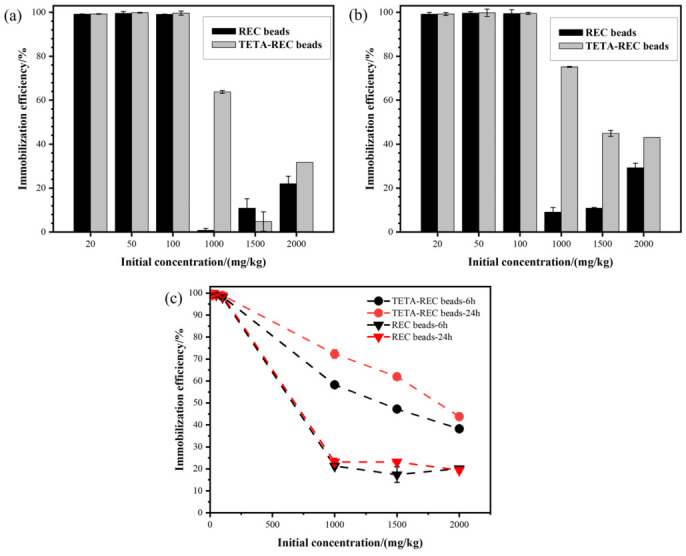
Effect of different concentrations of Cd(II)-contaminated soil. (**a**) Immobilization efficiency for water soluble-state Cd(II) at 6 h; (**b**) immobilization efficiency for water soluble-state Cd(II) at 24 h; (**c**) immobilization efficiency for available-state Cd(II) (Dosage of beads = 0.05 g, soil/solution = 1 g/5 mL).

Figure 3 illustrates the passivation efficiencies of REC beads and TETA-REC beads toward water-soluble and available Cd(II) in soil at different initial Cd(II) concentrations. Specifically, both materials exhibited relatively high passivation efficiencies after 24 h, and the immobilization process gradually reached equilibrium. At environmentally relevant Cd(II) concentrations (20–100 mg/kg), both REC beads and TETA-REC beads consistently exhibited high immobilization efficiencies, with only relatively small differences observed among the tested concentration levels. This indicates that both materials possess stable and effective Cd(II) immobilization capacities under low-to-moderate contamination conditions, making it difficult to comprehensively evaluate the enhancement effect introduced by TETA modification. Higher Cd(II) concentrations were further employed to better distinguish the immobilization behaviors of the two materials and to investigate their immobilization mechanisms under severe contamination conditions. However, their responses to increasing Cd(II) concentration differed significantly. For TETA-REC beads, the passivation efficiency decreased with increasing initial Cd(II) concentration, whereas REC beads showed the opposite trend, with improved efficiency under higher Cd(II) concentrations. When the initial Cd(II) concentration was 1000 mg/kg, the passivation efficiencies of TETA-REC beads for water-soluble and available Cd(II) reached 75.15% and 72.31%, respectively, which were significantly higher than those of REC beads (9.05% and 23.16%). This enhanced performance is mainly attributed to the abundant –NH_2_ groups introduced by TETA modification, which are distributed on the surface and within the interlayer of the clay mineral. These amino groups can form stable coordination complexes with Cd^2+^ through the donation of lone-pair electrons from nitrogen atoms to the empty orbitals of Cd^2+^, thereby significantly enhancing the chemical immobilization capacity. In addition, the intercalation of TETA molecules improves the accessibility of interlayer spaces and promotes cation-exchange reactions between Ca^2+^ and Cd^2+^, resulting in a synergistic immobilization mechanism involving both complexation and ion exchange. When the Cd(II) concentration increased to 2000 mg/kg, the passivation efficiencies of TETA-REC beads decreased to 42.99% for water-soluble Cd(II) and 43.82% for available Cd(II). Under these conditions, the coordination sites gradually approached saturation, and part of the Cd^2+^ could no longer be effectively complexed by amino groups, relying instead on weaker physical adsorption or electrostatic interactions. Meanwhile, intensified competition among Cd^2+^ and other cations under high-concentration conditions inhibited Ca^2+^ release and Cd^2+^ exchange processes, thereby increasing the proportion of reversible binding and reducing the overall immobilization efficiency. In contrast, unmodified REC beads exhibited relatively low passivation efficiency at low Cd(II) concentrations due to limited adsorption and exchange sites. However, at higher concentrations, the increased concentration gradient enhanced the driving force for ion exchange, resulting in an increase in the passivation efficiency of water-soluble Cd(II) from 9.05% to 29.26%. This suggests that unmodified REC can immobilize Cd(II) to some extent through ion exchange and physical adsorption under high-concentration conditions, although its overall capacity and stability remain inferior to those of TETA-modified materials. These results indicate that the organic modification of REC significantly enhances chemical complexation through the introduction of abundant –NH_2_ groups while improving interlayer accessibility and material dispersibility. This results in a dual-mechanism immobilization advantage at relatively low Cd(II) concentrations, whereas the efficiency decline at high concentrations is mainly limited by the saturation of coordination sites.

### 2.4. Effect of REC Microsphere Dosage on Cd(II) Passivation

**Figure 4:**
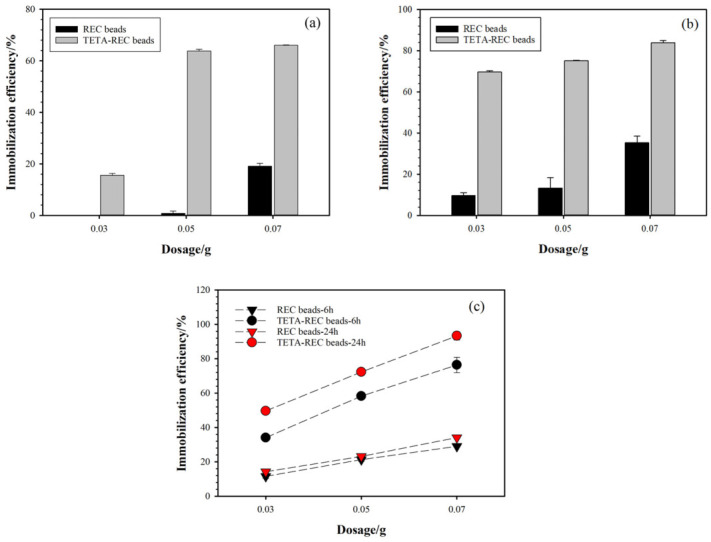
Effect of passivators dosages. (**a**) Immobilization efficiency for water soluble-state Cd(II) at 6 h, (**b**) immobilization efficiency for water soluble-state Cd(II) at 24 h, (**c**) immobilization efficiency for available-state Cd(II) (concentration of Cd(II)-contaminated soil: 1000 mg/kg, dosage of beads = 0.05 g, soil/solution = 1 g/5 mL).

Figure 4 shows the passivation efficiencies of REC beads and TETA-REC beads toward water-soluble and available Cd(II) in soil under different dosage and reaction time conditions. Overall, the passivation efficiency increased with increasing dosage for both materials, while TETA-REC beads consistently exhibited significantly higher immobilization performance than REC beads. For water-soluble Cd(II), when the passivation time was 6 h (Figure 4a), increasing the dosage from 0.03 g to 0.07 g led to only a slight increase in the passivation efficiency of REC beads, from 0 to 19.10%. In contrast, the passivation efficiency of TETA-REC beads increased markedly from 15.57% to 66.02% over the same dosage range. When the passivation time was extended to 24 h (Figure 4b), the passivation efficiencies of REC beads increased from 9.64% to 35.25%, whereas those of TETA-REC beads further increased from 69.68% to 83.87%. These results indicate that TETA modification substantially enhanced the immobilization capacity of the microspheres toward water-soluble Cd(II), particularly under prolonged reaction conditions. Figure 4c presents the passivation efficiencies of the two microspheres toward available Cd(II) in soil. At a passivation time of 6 h, increasing the dosage from 0.03 g to 0.07 g increased the passivation efficiency of REC beads from 11.54% to 29.01%, while the efficiency of TETA-REC beads increased from 34.04% to 76.41%. After 24 h of passivation, the efficiencies of REC beads increased only from 4.25% to 34.10%, whereas TETA-REC beads achieved significantly higher efficiencies ranging from 49.60% to 93.33%. The results demonstrate that REC beads exhibited relatively limited passivation ability toward both water-soluble and available Cd(II). Even under relatively high dosage and prolonged reaction time conditions, the passivation efficiency remained below 40%. Moreover, under short reaction time and low-dosage conditions, REC beads even promoted the release of water-soluble Cd(II) in soil. In comparison, TETA-REC beads exhibited substantially superior immobilization performance. The prolonged passivation process provided sufficient time for Cd(II) species to diffuse into the microspheres and become effectively immobilized through complexation and ion-exchange interactions. For example, when 0.07 g of TETA-REC beads was applied for 24 h, the passivation efficiencies toward water-soluble and available Cd(II) reached 83.87% and 93.33%, respectively. Although the dosage applied in this laboratory-scale study was relatively high, the primary objective was to evaluate the feasibility and immobilization mechanisms of REC-based microspheres under controlled experimental conditions. Given the high Cd(II) concentrations employed in this experiment, it was also necessary to systematically investigate the relationship between dosage and passivation performance within a relatively high dosage range. In addition, the bead structure may reduce the risk of secondary dispersion commonly associated with powdered passivators and facilitate material handling during soil remediation processes. Nevertheless, further studies are still required to optimize the application dosage, improve active-site utilization efficiency, and evaluate the long-term economic feasibility and field-scale applicability of the microspheres under realistic agricultural conditions. These findings further confirm that TETA modification significantly improved the Cd(II) immobilization performance of REC microspheres and enhanced their potential for the remediation of Cd-contaminated soils.

### 2.5. Effect of pH on REC Microsphere Passivation

Considering that Cd(II) can precipitate as Cd(OH)_2_ under alkaline conditions, the soil pH in this study was controlled within the range of 3–6 to avoid precipitation interference. As shown in Table 1, when the pH values were 3, 4, 5, and 6, the concentrations of water-soluble Cd(II) in the control group were 86.98, 88.37, 70.42, and 19.19 mg/L, respectively, while the corresponding concentrations of available Cd(II) were 40.73, 52.97, 52.88, and 68.43 mg/L. These results indicate that under strongly acidic conditions, the concentration of water-soluble Cd(II) increased significantly, whereas the concentration of available Cd(II) decreased correspondingly. This suggests that acidic environments promote the transformation of Cd(II) from exchangeable forms to more mobile water-soluble forms. When the pH increased to 6, the concentrations of both forms tended to stabilize, likely because this value is close to the original soil background pH (approximately 6.6), thereby enhancing the buffering capacity of the soil system.

**Figure 5:**
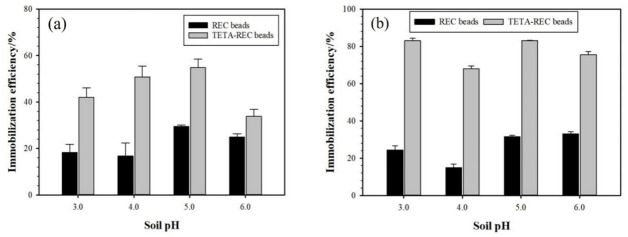
The effect of soil pH. (**a**) Immobilization efficiency for water soluble-state Cd(II), (**b**) immobilization efficiency for available Cd(II) (concentration of Cd(II)-contaminated soil: 1000 mg/kg, dosage of bead: 0.05 g, T = 24 h, soil/solution = 1 g/5 mL).

As shown in Figure 5a, the passivation efficiency of both REC beads and TETA-REC beads for water-soluble Cd(II) initially increased and then decreased with increasing pH, reaching a maximum at pH 5. At this point, TETA-REC beads achieved a passivation efficiency of 54.81%, which was significantly higher than that of REC beads (29.44%). In contrast, Figure 5b demonstrates that the passivation efficiency for available Cd(II) was less sensitive to pH variation, although both materials exhibited their lowest efficiencies at pH 4, with values of 68.03% for TETA-REC beads and 14.90% for REC beads. Across the tested pH range, TETA-REC beads consistently showed higher immobilization efficiency for available Cd(II) than for water-soluble Cd(II), maintaining a relatively stable efficiency of approximately 80%, whereas the maximum efficiency of REC beads remained below 35%. This difference is mainly attributed to the pH-dependent speciation of Cd(II) and the surface properties of the materials. Under weakly acidic conditions (pH 4–5), Cd^2+^ predominantly exists as free ions, facilitating interaction with interlayer cation-exchange sites and active functional groups, while the abundant –NH_2_ groups in TETA-REC beads promote the formation of stable coordination complexes, thereby enhancing binding capacity even under acidic conditions. As the pH approaches neutrality, Cd^2+^ gradually hydrolyzes to form hydroxide species, reducing its direct interaction with the material surface, and the increased buffering capacity of the soil further weakens the driving force for cation exchange. These results indicate that weakly acidic conditions are more favorable for Cd(II) immobilization, and that TETA modification significantly enhances passivation efficiency through the synergistic effects of complexation and ion exchange under varying pH conditions.

### 2.6. Effects of Freeze–Thaw Cycles

**Figure 6:**
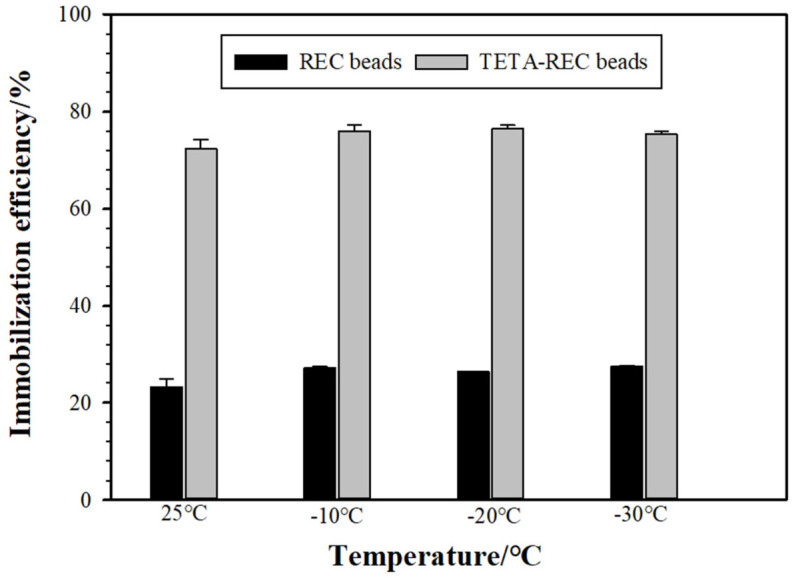
The effect of freeze–thaw with different temperatures for immobilization efficiency of available Cd(II) (concentration of Cd(II)-contaminated soil: 1000 mg/kg, dosage of bead: 0.05 g, T = 24 h, soil/solution = 1 g/5 mL).

**Figure 7:**
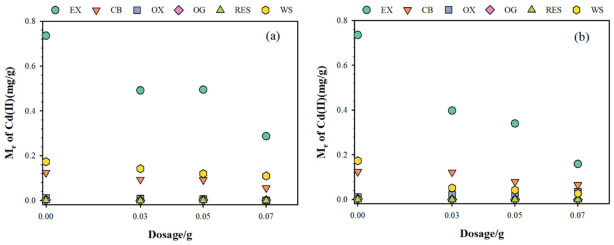
Content of different Cd(II) state in soil after immobilization (**a**) REC beads, (**b**) TETA-REC beads (Mass of extracted soil: 0.5 g, T = 24 h, EX: exchangable state, CB: carbonate state, OX: Fe–Mn oxidation state, OG: organic state, RES: residual state, WS: water-soluble state, M_e_: mass of Cd(II) per 1 g of soil, concentration of Cd(II)-contaminated soil: 1000 mg/kg).

Figure 6 illustrates the variation in passivation efficiency of REC beads and TETA-REC beads for available Cd(II) in soil under different freeze–thaw cycles. The results indicate that freeze–thaw processes exerted minimal influence on the passivation performance of both materials, reflecting their favorable environmental stability. Specifically, the passivation efficiency of REC beads remained relatively constant at approximately 27%, whereas that of TETA-REC beads was consistently maintained around 77%, nearly three times higher than that of REC beads, demonstrating that TETA modification markedly enhances both complexation capacity and structural stability. From a mechanistic perspective, freeze–thaw cycles induce repeated expansion and contraction of the soil matrix, leading to changes in pore structure and water distribution, which may influence the contact efficiency between the adsorbent and Cd^2+^. However, the abundant –NH_2_ functional groups on TETA-REC beads facilitate the formation of stable coordination bonds and electrostatic interactions with Cd^2+^, thereby sustaining effective immobilization and mitigating the loss of active sites under temperature stress. In contrast, REC beads primarily rely on cation exchange and physical adsorption, which involve weaker interactions and consequently result in lower immobilization efficiency. In addition, the results show that freeze–thaw cycles did not significantly affect the leaching behavior of available Cd(II) in the control soil, with concentrations remaining stable at approximately 65 mg/L under all tested conditions, indicating that the influence of freeze–thaw processes on Cd(II) speciation is considerably weaker than that of pH variation (see Table 1 and Figure 7). Overall, the results demonstrate that TETA-REC beads exhibit strong immobilization capability and environmental resilience under freeze–thaw conditions.

### 2.7. Speciation and Extraction of Cd

Since the microspheres embedded in soil are difficult to completely dissolve during chemical extraction, the microspheres were first separated from the soil after passivation treatment to ensure the accuracy of Cd(II) speciation analysis. Subsequently, the remaining soil samples were subjected to sequential extraction based on the Tessier method to determine the distribution of Cd(II) among different geochemical fractions. The variation in Cd(II) speciation under different microsphere dosages is illustrated using a scatter plot in Figure 7. In the contaminated soil with an initial Cd(II) concentration of 1000 mg/kg, the measured total Cd content was 1.05 mg/g, which is highly consistent with the theoretical value, indicating the reliability of the experimental procedure. In the untreated soil, Cd(II) was mainly present in exchangeable (EX), water-soluble (WS), and carbonate-bound (CB) fractions, whereas the contents of oxide-bound (OX), organic matter-bound (OM), and residual (RES) fractions were relatively low. With the increase in microsphere dosage from 0 to 0.07 g, significant changes in Cd(II) speciation were observed. For REC beads, the total extractable Cd content decreased to 0.46 mg/g, with the EX, CB, and WS fractions decreasing to 0.29, 0.06, and 0.11 mg/g, respectively. In comparison, TETA-REC beads exhibited a more pronounced immobilization effect, reducing the total Cd content to 0.29 mg/g. Correspondingly, the EX, CB, and WS fractions decreased to 0.16, 0.07, and 0.03 mg/g, respectively, while the OX fraction slightly increased to 0.03 mg/g, indicating that part of the Cd(II) was transformed into relatively stable forms. The EX and WS fractions are considered highly mobile and bioavailable forms with elevated environmental risk. Their substantial reduction suggests that the potential risk of Cd(II) remobilization and plant uptake in the soil–water system was significantly decreased after microsphere treatment. Although the CB fraction can still be released under acidic conditions, the increase in OX, OM, and RES fractions indicates enhanced stabilization of Cd(II) in the soil matrix. Compared with REC beads, TETA-REC beads demonstrated a stronger ability to transform Cd(II) from labile fractions to more stable forms. This improvement is mainly attributed to the introduction of abundant –NH_2_ and –NH– functional groups through TETA modification, which provide high-density coordination sites capable of forming stable complexes with Cd^2+^. As a result, TETA-REC beads not only significantly reduce the concentrations of EX and WS fractions but also promote the transformation of Cd(II) into more stable fractions such as OX, thereby improving the resistance of the immobilized Cd to environmental disturbances such as soil acidification. This enhanced stabilization performance suggests that TETA-REC beads have considerable potential as an effective remediation material for the long-term safe utilization of Cd-contaminated agricultural soils. Furthermore, based on the change in Cd(II) content in soil before and after passivation treatment, the immobilization capacity of the microspheres can be calculated using Equation (1):

(1) $$Q_{e}=\frac{M_{0}-M_{e}}{M}$$

where *Q_e_* represents the Cd(II) passivation capacity of the microspheres (mg/g), *M_0_* is the initial Cd(II) mass (mg) in 1 g of contaminated soil, *M_e_* is the residual Cd(II) mass (mg) in the treated soil, and *M* is the dosage of microspheres added (0.07 g in this experiment).

According to the calculation results, the Cd(II) passivation capacity of REC beads was 8.43 mg/g, whereas that of TETA-REC beads reached 10.86 mg/g. This result further confirms that TETA modification significantly enhances the Cd(II) immobilization performance of the REC microspheres.

### 2.8. Soil Microbial Community Structure and Composition

#### 2.8.1. Richness and Diversity of Microbial Communities

**Figure 8:**
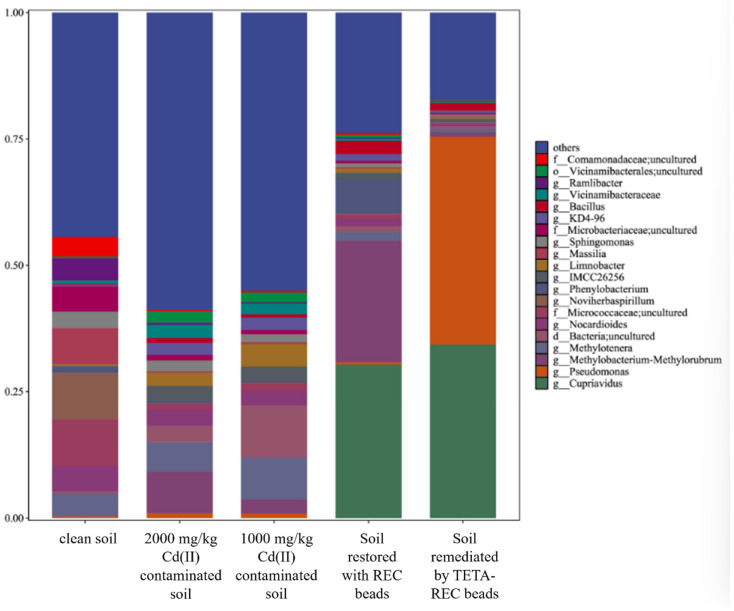
Microbial population histogram.

According to the horizontal community composition results (Figure 8), the structure of soil microbial communities changed significantly under different treatments, indicating that Cd(II) contamination and the introduction of remediation materials exerted a substantial influence on the soil microbial ecosystem. In the uncontaminated soil, the microbial community exhibited a rich and balanced composition, with relatively high abundances of common environmental bacteria such as *Bacillus*, *Sphingomonas*, *Massilia*, and *Phenylobacterium*. These genera represent typical organic matter–degrading bacteria, nutrient cycling microorganisms, and plant growth–promoting bacteria in healthy soils. This community structure reflects the high diversity and functional redundancy of natural soil ecosystems, where microbial taxa maintain strong metabolic complementarity and ecological interactions. However, after Cd(II) contamination, the microbial community structure became significantly disrupted. In soil contaminated with 2000 mg/kg Cd(II), community diversity decreased markedly and the overall structure became more homogenized. Originally dominant neutral or metal-sensitive bacteria (such as *Bacillus* and *Sphingomonas*) were strongly inhibited, whereas the relative abundances of heavy metal-tolerant genera, including *Cupriavidus* and *Pseudomonas*, increased substantially. This shift indicates that high concentrations of Cd(II) imposed strong ecological selection pressure on soil microorganisms, allowing only species possessing metal tolerance mechanisms and oxidative stress resistance to survive. In soils contaminated with 1000 mg/kg Cd(II), the disturbance to community structure was relatively less severe; however, microbial diversity was still lower than that of the original soil, reflecting the detrimental impact of Cd(II) pollution on the soil microbial ecological network. Following the addition of REC microspheres, the soil microbial community gradually began to recover, and the dominant taxa shifted from a single metal-tolerant population toward a more multifunctional and symbiotic community structure. In soil treated with REC beads, genera such as *Pseudomonas*, *Massilia*, and *Sphingomonas* reappeared and their relative abundances increased noticeably, suggesting that the application of REC beads effectively reduced the bioavailability and toxicity of Cd(II). The layered structure of the clay mineral combined with the sodium alginate matrix forms a porous microenvironment that can serve as a favorable attachment and colonization site for microorganisms. This structure not only provides physical protection against metal stress but also improves the microhabitat stability of soil microorganisms. In addition, the ion-exchange and adsorption properties of the material can partially immobilize Cd(II), thereby reducing direct exposure of microorganisms to toxic metal ions and facilitating microbial community reconstruction. Compared with REC beads, remediation using TETA-REC beads showed a more pronounced effect on microbial community recovery. In this system, metal-resistant genera such as *Cupriavidus* and *Pseudomonas* remained dominant, while multifunctional bacteria including *Massilia* and *Bacillus* were also detected in higher abundance. The resulting community composition was closer to that of the natural soil ecosystem. This enhanced recovery can be attributed to the polyamine functional groups introduced by TETA molecules, which can form stable coordination complexes with Cd(II), significantly reducing the free Cd(II) activity in soil solution and thereby alleviating metal stress. At the same time, the amine-functionalized surface improves the biocompatibility and surface charge characteristics of the microspheres, promoting bacterial adhesion and biofilm formation and enabling stable microbial colonization at the remediation interface. The enrichment of genera such as *Cupriavidus* and *Pseudomonas*, which possess strong metal resistance and electron transfer capabilities, may further enhance Cd immobilization through mechanisms such as extracellular electron transport and extracellular polymeric substance secretion, thereby contributing to the overall remediation process.

#### 2.8.2. Species and Functional Distribution in Soil Samples

**Figure 9:**
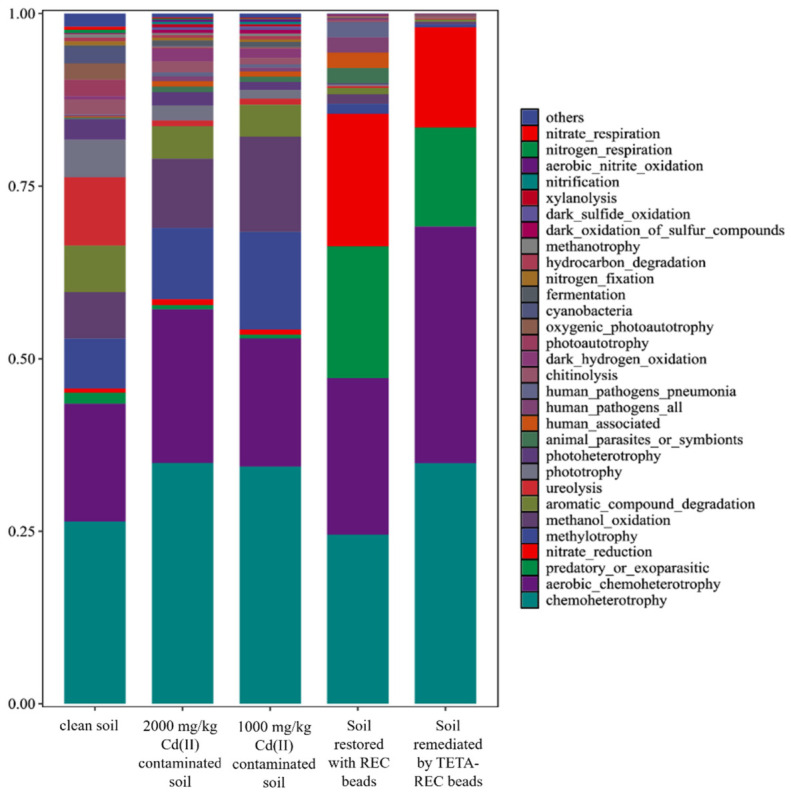
Functional richness histogram.

The functional prediction analysis (Figure 9) further revealed that Cd(II) contamination and subsequent remediation significantly affected the metabolic functions of soil microbial communities. Heavy metal pollution suppressed core metabolic functions, whereas remediation treatments promoted the recovery of energy metabolism and biogeochemical cycling functions.

In uncontaminated soils, microbial functional profiles were diverse and mainly included aerobic heterotrophy, chemoheterotrophy, nitrogen cycle–related processes (such as nitrification, nitrogen respiration, and nitrogen fixation), and organic carbon degradation pathways (including aromatic compound degradation and methanol oxidation). The coexistence of these metabolic functions indicates that natural soils maintain coordinated carbon and nitrogen cycling processes, supported by well-developed redox systems and energy metabolism pathways within the microbial community. After Cd(II) contamination, the microbial functional composition changed markedly. In soils contaminated with 2000 mg/kg Cd(II), the functions related to aerobic chemoheterotrophy and nitrification decreased significantly, while nitrate respiration and nitrogen respiration functions increased. This shift indicates a transition in microbial metabolism from aerobic pathways toward facultative or anaerobic metabolic processes. This metabolic transition is likely caused by the inhibition of the oxidative respiratory chain and key enzymatic systems, such as NADH dehydrogenase and cytochrome complexes, by high concentrations of Cd(II). As a result, microorganisms rely on alternative energy pathways such as nitrate respiration to maintain energy supply. At the same time, reductive metabolic processes such as methanotrophy and fermentation were enhanced, suggesting that anaerobic or metal-reducing microorganisms may become dominant under heavy metal stress. In soils contaminated with 1000 mg/kg Cd(II), although the reduction in overall functional diversity was less pronounced, key ecological functions such as nitrogen fixation and organic matter degradation were still significantly suppressed. This indicates that Cd(II) pollution exerts persistent and concentration-dependent inhibition on microbial metabolic networks. After remediation treatment, the functional profiles of soil microorganisms showed a clear recovery trend. In soils treated with REC beads, the relative abundances of chemoheterotrophy and aerobic chemoheterotrophy increased significantly, while nitrate respiration and nitrogen respiration functions decreased. These results suggest that the addition of REC beads alleviated metal toxicity and improved oxygen availability within the soil microenvironment, thereby promoting the recovery of aerobic metabolic processes. In addition, the enhanced functions associated with organic carbon degradation, including aromatic compound degradation and methanol oxidation, indicate that the REC bead system contributes to the restoration of microbial capacity for decomposing complex organic matter. In soils treated with TETA-REC beads, microbial functional recovery was even more pronounced. The proportion of aerobic chemoheterotrophy further increased, reflecting the enhanced Cd immobilization capability of the TETA-modified material, which effectively reduced metal toxicity and created a more favorable habitat for aerobic microorganisms. The reactivation of nitrification and nitrogen fixation functions, accompanied by decreases in fermentation and nitrate respiration processes, indicates that the soil nitrogen cycle gradually returned toward equilibrium. Microbial metabolism therefore shifted from anaerobic reduction pathways toward more stable aerobic oxidation processes. The resurgence of functional groups involved in methanotrophy and aromatic compound degradation further suggests that the remediation system facilitated the restoration of carbon cycling processes and microbial electron transport activity. These results indicate that Cd(II) contamination caused structural disruption and functional degradation of soil microbial communities. Application of both REC beads and TETA-REC beads facilitated ecological reconstruction of the microbial community, with TETA-REC beads showing the most pronounced regulatory and recovery effects.

### 2.9. Post-Reaction Characterization and Mechanism Analysis of REC Microspheres

**Figure 10:**
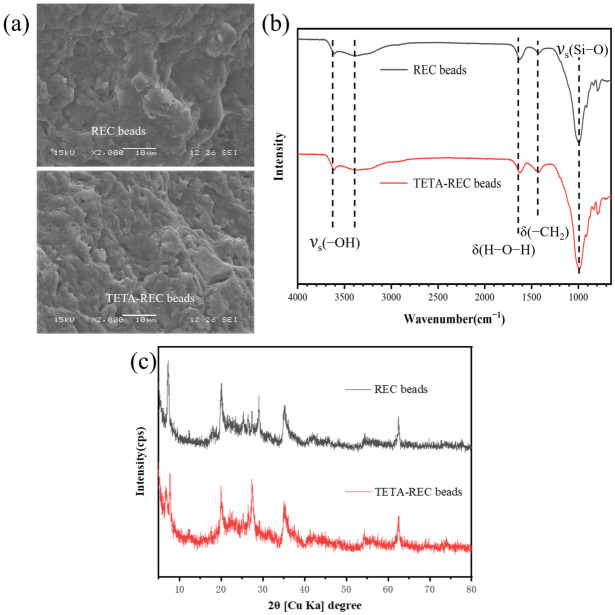
(**a**) The SEM of REC beads after immobilization. (**b**) The FTIR spectrum of REC beads after immobilization. (**c**) The XRD results of REC beads after immobilization.

After the passivation experiments, the REC microspheres were separated from the soil and their surface morphology was characterized, as shown in Figure 10a. The results indicate that the microspheres retained the typical layered structure of the clay mineral after passivation. However, the surface morphology became relatively rough and partially covered by fine particles. This phenomenon is likely attributed to the deposition or attachment of soil components onto the surface of the microspheres during the passivation process, which partially filled the pores and depressions and resulted in a relatively compact surface structure. For TETA-REC beads, some dried REC particles and clay lamellae were still observed on the microsphere surface, whereas the surface of REC beads appeared relatively smoother and more intact. This observation suggests that the introduction of TETA molecules may cause slight structural disturbance on the surface of the composite material, although the overall morphology remained stable.

To further investigate the structural stability of the materials after passivation, the microspheres were ground into powder and characterized by FTIR (Figure 10b) and XRD (Figure 10c). The FTIR spectra showed that the main functional groups of the microspheres remained largely unchanged compared with those before passivation, indicating that the overall chemical structure of the materials was well preserved during the remediation process. However, the absorption peak intensities of TETA-REC beads at approximately 3380 cm^−1^ (O–H stretching vibration) and 1640 cm^−1^ (H–O–H bending vibration) were significantly enhanced after passivation, suggesting that the material adsorbed more water molecules during the reaction process. This phenomenon may be related to the strong coordination interactions between TETA functional groups and Cd(II), which promote hydration around the complexation sites. The XRD results further demonstrated that the crystal structure of REC remained stable after passivation. The characteristic diffraction peaks corresponding to the layered structure were still clearly observed, indicating that the embedding process and remediation reaction did not destroy the lattice structure of the clay mineral. The calculated d_002_ values of REC beads and TETA-REC beads after reaction were 1.23 nm and 1.34 nm, respectively, which are close to those before passivation, confirming that the interlayer structure of the material remained essentially stable.

**Figure 11:**
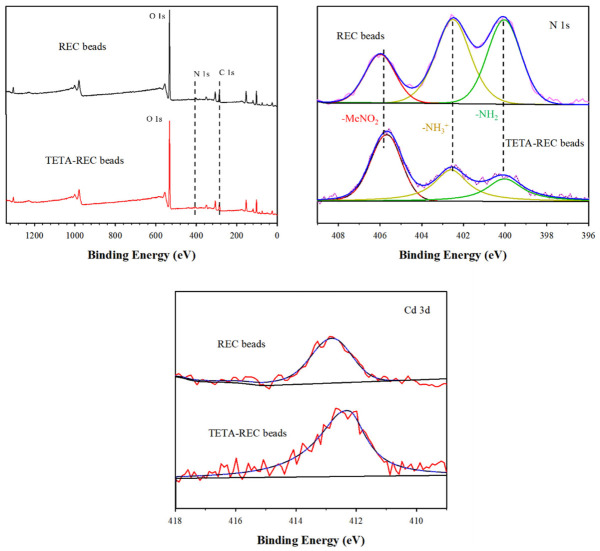
The XPS results of REC beads after immobilization.

X-ray photoelectron spectroscopy (XPS) analysis (Figure 11) further confirmed the presence of Cd(II) on the surface of both types of microspheres, indicating their active involvement in Cd(II) immobilization during the passivation process. Combined with the morphological and structural characterization results, the passivation mechanism of Cd(II) by REC-based microspheres can be summarized as follows: Cd^2+^ is initially immobilized through cation exchange with interlayer cations (e.g., Ca^2+^) in the clay mineral structure; subsequently, physical adsorption occurs via the sodium alginate matrix and the porous surface of REC, which provide abundant binding sites for Cd^2+^ accumulation; additionally, in the case of TETA-REC beads, the introduced –NH_2_ functional groups further enhance Cd(II) immobilization through coordination complexation by donating lone-pair electrons to form stable metal–ligand complexes, thereby significantly improving the binding strength and long-term stability of Cd.

**Figure 12:**
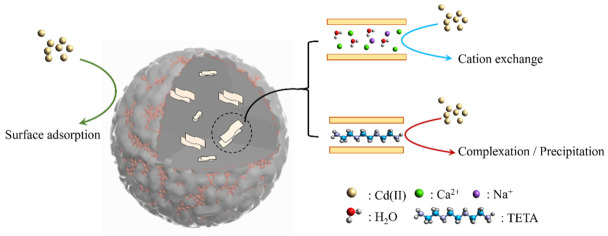
Passivation mechanism diagram of REC beads.

As summarized in Figure 12, the sodium alginate encapsulation structure provides REC microspheres with enhanced structural stability and resistance to environmental perturbation, while enabling synergistic immobilization of Cd(II) through multiple mechanisms. Under near-neutral pH conditions (pH ≈ 6–7), Cd(II) predominantly exists as free Cd^2+^, while hydrolyzed species are thermodynamically negligible, as indicated by the low hydrolysis constants:

Cd^2+^ + H_2_O ⇌ CdOH^+^ + H^+^ (logK ≈ −10.1)

CdOH^+^ + H_2_O ⇌ Cd(OH)_2_(aq) + H^+^ (logK ≈ −19 to −20)

Thus, Cd^2+^ is the dominant aqueous species in the system [33]. For TETA-modified REC microspheres, amine groups undergo protonation according to:

R-NH_2_ + H^+^ ⇌ R-NH_3_^+^ (pKa ≈ 9–10 for aliphatic amines)

At near-neutral pH, this equilibrium results in partial protonation, leading to the coexistence of protonated and neutral nitrogen species [34]. Consequently, a significant fraction of –NH_2_ groups remains available for coordination with Cd^2+^. Cd(II) can further form stable inner-sphere complexes with amine ligands:

Cd^2+^ + L ⇌ CdL^2+^ (logK ≈ 4–8 for polyamine ligands)

Previous studies have confirmed that polyamine-functionalized materials exhibit strong binding affinity toward Cd^2+^ due to multidentate coordination and the presence of multiple nitrogen donor sites [35]. Although protonation introduces electrostatic competition, Cd binding is not suppressed because (i) unprotonated amine sites remain accessible and (ii) multidentate chelation in TETA significantly enhances complex stability. Cd immobilization in the TETA-REC system is governed by a coupled mechanism involving ion exchange, surface adsorption, and inner-sphere complexation, not simple inhibition by amine protonation. This interpretation is supported by the higher immobilization efficiency observed for TETA-REC beads compared with unmodified REC beads, confirming the active role of amine functional groups in Cd uptake. TETA-REC beads retain the intrinsic ion-exchange and adsorption capacity of the clay-alginate matrix while introducing a strong Cd-amine coordination pathway. This synergistic effect promotes the transformation of Cd(II) from exchangeable and water-soluble fractions toward more stable forms, such as oxidizable or organically bound species. Cd mobility and bioavailability are significantly reduced, enabling long-term stabilization in contaminated agricultural soils.

## 3. Materials and Methods

### 3.1. Materials

All reagents used in this experiment were of analytical grade (AR) or chemically pure grade (GR). Specifically, cadmium nitrate tetrahydrate (Cd(NO_3_)_2_·4H_2_O, AR), sodium alginate (C_6_H_7_NaO_6_, GR), sodium hydroxide (NaOH, AR), sulfuric acid (H_2_SO_4_, AR), anhydrous calcium chloride (CaCl_2_, AR), glacial acetic acid (CH_3_COOH, AR), magnesium chloride hexahydrate (MgCl_2_·6H_2_O, AR), sodium acetate (CH_3_COONa, AR), hydroxylamine hydrochloride (NH_2_OH·HCl, AR), nitric acid (HNO_3_, AR), hydrogen peroxide (H_2_O_2_, AR), ammonium acetate (CH_3_COONH_4_, AR), and hydrochloric acid (HCl, AR) were purchased from Sinopharm Chemical Reagent Co., Ltd. (Shanghai, China); triethylenetetramine (C_6_H_18_N_4_, AR) was purchased from Aladdin Biochemical Technology Co., Ltd. (Shanghai, China).

### 3.2. Preparation of TETA-REC

Rectorite (REC) used in this study was purchased from Zhongxiang Mingliu Co., Ltd. (Jingmen, China) and had a cation exchange capacity (CEC) of 44.91 mmol/g. To prepare triethylenetetramine-modified rectorite (TETA-REC), 3 g of REC was dispersed in 50 mL of ultrapure water and magnetically stirred for 30 min to ensure uniform dispersion. Subsequently, triethylenetetramine (TETA) was added at a molar ratio of TETA to the cation exchange capacity of REC of 2:1 to initiate the modification reaction. The suspension was then transferred to a thermostatic water bath at 70 °C and continuously stirred for 3 h. After the reaction, the mixture was centrifuged at 6000 rpm for 5 min, and the supernatant was discarded. The obtained solid was washed three times with ultrapure water to remove residual reagents, followed by drying and grinding to obtain TETA-REC.

### 3.3. Preparation of REC Microspheres (REC Beads and TETA-REC Beads)

For the preparation of microspheres, 6 g of REC or TETA-REC was dispersed in 44 mL of ultrapure water to form a suspension with a solid mass fraction of 12%. Sodium alginate (0.5 g, corresponding to a mass concentration of 1%) was then added, and the mixture was stirred at room temperature for 10 min. The suspension was subsequently heated in a 60 °C water bath and stirred for an additional 30 min until the sodium alginate was completely dissolved, forming a homogeneous viscous solution. The resulting solution was slowly dropped into a 3% CaCl_2_ solution using a dropper to induce ionic crosslinking and form gel beads. After stabilization, the beads were washed three times with ultrapure water and dilute nitric acid (HNO_3_) to remove residual CaCl_2_ from the surface. Finally, the beads were dried in an oven at 40 °C to obtain REC beads or TETA-REC beads.

### 3.4. Preparation of Cd(II)-Containing Soil Leachate

Original soil samples were collected from Guangdong Province, China. After collection, visible impurities such as stones, plant roots, and plastic debris were removed. The soil samples were then air-dried at room temperature for 3–5 days, ground using a mortar and pestle, and passed through a 100-mesh sieve to obtain homogenized soil for subsequent experiments.

To prepare Cd(II)-contaminated soils with different contamination levels, the pretreated soil was mixed with Cd(II) solutions at initial concentrations of 1000, 1500, and 2000 mg/L at a solid-to-liquid ratio of 1:1 (g/mL). The mixtures were thoroughly stirred using a magnetic stirrer to ensure uniform distribution of Cd(II). The samples were subsequently dried in an oven at 50 °C. After drying, the soils were crushed and ground to obtain contaminated soils with Cd(II) concentrations of approximately 1000, 1500, and 2000 mg/kg, respectively.

To investigate the effect of pH on the properties of Cd-contaminated soils, additional soil samples were prepared by mixing the pretreated soil with a 1000 mg/L Cd(II) solution at the same solid–liquid ratio (1:1, g/mL). The pH of the mixture was adjusted to 3, 4, 5, and 6 using 2 mol/L HNO_3_ or 2 mol/L NaOH. The treated mixtures were then dried at 50 °C, followed by crushing and grinding to obtain Cd(II)-contaminated soils under different pH conditions.

### 3.5. Cd(II) Passivation Experiment

Cd(II)-contaminated soil samples (1 g) with different contamination levels (1000, 1500, and 2000 mg/kg) were placed in 10 mL centrifuge tubes. REC microspheres were then added at different dosages (0, 0.03, 0.05, and 0.07 g), followed by the addition of 5 mL of deionized water. The mixtures were placed on a rotary mixer and reacted at room temperature under continuous mixing at 30 ± 2 rpm. The reaction times were set to 0, 5, 30, 60, 120, 240, 360, and 1440 min.

After the reaction, the samples were centrifuged at 6000 rpm, and the supernatants were collected and filtered through a 0.45 μm aqueous membrane filter. The filtrates were appropriately diluted, and the Cd(II) concentrations were determined using atomic absorption spectrometry (AAS). The passivated soil samples were subsequently dried and collected for further analysis.

### 3.6. Freeze–Thaw Cycle Experiment

Cd(II)-contaminated soil samples (1000 mg/kg Cd, REC microsphere dosage of 0.05 g) after 12 h of passivation were selected for the freeze–thaw cycle experiment. Deionized water (0.7 mL) was added to adjust the soil moisture content to approximately 70%. The samples were then placed in an ultra-low-temperature chamber and frozen at −10 °C, −20 °C, and −30 °C for 12 h, followed by thawing at 25 °C for 12 h, which constituted one freeze–thaw cycle. Each treatment underwent three freeze–thaw cycles.

After the freeze–thaw treatment, 5 mL of 0.01 mol/L CaCl_2_ solution was added to the soil samples, and the mixtures were shaken on a rotary mixer for 3 h to extract available Cd(II) from the soil. The samples were subsequently centrifuged at 6000 rpm, and the supernatants were collected and filtered through a 0.45 μm membrane filter. The filtrates were diluted appropriately, and the Cd(II) concentrations were determined using atomic absorption spectrometry. All experiments were conducted with control and parallel samples to ensure the accuracy and reproducibility of the experimental results. The potential interference of Ca ions on Cd quantification by atomic absorption spectroscopy was evaluated based on previous experimental studies. Waterlot et al. [36] systematically investigated the influence of CaCl_2_ in the CaCl_2_ extraction method on graphite furnace atomic absorption spectrometry (GFAAS). Their results demonstrated that no significant effect on the Cd signal was observed at low CaCl_2_ concentrations, whereas noticeable interference only occurred at substantially elevated matrix levels. Wu et al. [37] reported that Ca(NO_3_)_2_ up to 0.02% (*w*/*v*) had no measurable impact on Cd determination by flame AAS.

### 3.7. Extraction Experiment of Cd(II) Species in Soil Leachate

In this study, water leaching, calcium chloride extraction, and the Tessier sequential extraction method were employed to evaluate the occurrence forms of Cd(II) in soil. Water leaching was used to determine the concentration of water-soluble Cd(II) in the soil, and the passivation efficiency of REC microspheres was calculated based on this value to assess their immobilization performance.

The passivation efficiency was calculated using the following equation:

(2) $$E_{i}=\frac{C_{0}-C_{i}}{C_{0}}\times 100\%$$

where *E_i_* represents the passivation efficiency of REC microspheres for Cd(II) (%); *C_0_* is the concentration of water-soluble Cd(II) in untreated contaminated soil (mg/L); and *C_i_* is the concentration of water-soluble Cd(II) in the contaminated soil after treatment with REC microspheres for a certain period (mg/L).

For Cd(II)-contaminated soils treated with REC microspheres, the calcium chloride extraction method was further used to determine the concentration of available Cd(II), which was applied to evaluate the effects of external environmental factors (e.g., freeze–thaw cycles and pH variation) on the passivation performance. In addition, the Tessier sequential extraction procedure was conducted to analyze the distribution of Cd(II) among different chemical fractions in the soil before and after passivation treatment, following the method described by Tessier et al. [38]. The results were used to elucidate the immobilization mechanism of Cd(II) by REC microspheres.

## 4. Conclusions

In this study, sodium alginate-based REC microspheres and TETA-modified REC microspheres were successfully fabricated and applied for the immobilization of Cd(II) in contaminated soils. Structural characterization confirmed that the encapsulation process preserved the layered structure of the clay mineral, while TETA modification introduced abundant amino functional groups that strengthened interactions with Cd(II). Both materials exhibited rapid and stable immobilization performance, with TETA-REC beads consistently demonstrating superior efficiency under varying environmental conditions; moreover, the immobilization efficiency increased with dosage and reached optimal performance under weakly acidic conditions. Mechanistic analysis revealed that Cd(II) immobilization was governed by the synergistic effects of cation exchange, physical adsorption, and coordination complexation, with the amino groups introduced by TETA providing additional coordination sites that enhanced Cd stabilization and facilitated its transformation from labile fractions to more stable forms in soil. In addition, the REC microsphere system showed good structural stability and resistance to environmental disturbances such as freeze–thaw cycles, while also promoting the recovery of soil microbial community structure and metabolic functions, thereby mitigating the ecological toxicity of Cd contamination. These results demonstrate that TETA-REC microspheres represent an effective and environmentally stable strategy for Cd(II) immobilization, with promising potential for the sustainable remediation and safe utilization of heavy metal-contaminated farmland; however, further studies on long-term field applications and multi-metal systems are needed to fully evaluate their practical applicability in complex contaminated soils.

## Figures and Tables

**Table 1:** The dissolution of water soluble-Cd(II) and available state-Cd(II) in soil at different pH.

Different pH Values	3	4	5	6
Blank sample water-soluble Cd(II) concentration/(mg/L)	86.98	88.37	70.42	19.19
Available Cd(II) concentration in blank samples/(mg/L)	40.73	52.97	52.88	68.43

## Data Availability

The raw data supporting the conclusions of this article will be made available by the authors on request.
